# Clinical validation of a smartphone-based handheld fundus camera for the evaluation of optic nerve head

**DOI:** 10.5935/0004-2749.20210080

**Published:** 2025-08-21

**Authors:** Carolina C. Titoneli, Marcio S. Filho, Diego Lencione, Flavio Pascoal Vieira, José Augusto Stuchi, Jayter S. Paula

**Affiliations:** 1 Faculdade de Medicina de Ribeirão Preto, Universidade de São Paulo, Ribeirão Preto, SP, Brazil; 2 Fundação PIO XII, Barretos, SP, Brazil; 3 Phelcom Technologies, São Carlos, SP, Brazil

**Keywords:** Photography/instrumentation, Smartphone, Optic nerve, Telemedicine, Fotografia/instrumentação, Smartphone, Nervo óptico, Telemedicina

## Abstract

**Purpose:**

To compare the quality of retinal images captured with a smartphone-based, handheld fundus camera with that of retinal images captured with a commercial fundus camera and to analyze their agreement in determining the cup-to-disc ratio for a cohort of ophthalmological patients.

**Methods:**

A total of 50 patients from a secondary ophthalmic outpatient service center underwent a bilateral fundus examination under mydriasis with a smartphone-based, handheld fundus camera and with a commercial fundus camera (4 images/patient by each). Two experienced ophthalmologists evaluated all the fundus images and graded them on the Likert 1-5 scale for quality. Multivariate regression analyses was then performed to evaluate the factors associated with the image quality. Two masked ophthalmologists determined the vertical cup-to-disc ratio of each fundus image, and both the intraobserver (between devices) and interobserver agreement between them was calculated.

**Results:**

Ninety-eight images from 49 patients were processed in this study for their quality analysis. Ten images from five patients (four from commercial fundus camera and one from smartphone-based, handheld fundus camera) were not included in the analyses due to their extremely poor quality. The medians [interquartile interval] of the image quality were not significantly different between those from the smartphone-based, handheld fundus camera and from the commercial fundus camera (4 [4-5] versus 4 [3-4] respectively, p=0.06); however, both the images captured with the commercial fundus camera and the presence of media opacity presented a significant negative correlation with the image quality. Both the intraobserver [intraclass correlation coefficient (ICC)=0.82, p<0.001 and 0.83, p<0.001, for examiners 1 and 2, respectively] and interobserver (ICC=0.70, p=0.001 and 0.81; p<0.001, for smartphone-based handheld fundus camera and commercial fundus camera, respectively) agreements were excellent and statistically significant.

**Conclusions:**

Our results thus indicate that the smartphone-based, handheld fundus camera yields an image quality similar to that from a commercial fundus camera, with significant agreement in the cup-to-disc ratios between them. In addition to the good outcomes recorded, the smartphone-based, handheld fundus camera offers the advantages of portability and low-cost to serve as an alternative for fundus documentation for future telemedicine approaches in medical interventions.

## Abstract (pt)

**Objetivo:**

Comparar a qualidade das imagens da retina capturadas com um retinógrafo portátil acoplado a um smartphone com aquelas adquiridas com um retinógrafo comercial padrão e analisar a concordância na determinação da relação escavação/ cabeça do nervo óptico em um coorte de pacientes de um serviço oftalmológico.

**Métodos:**

Cinquenta pacientes de um serviço oftalmológico secundário foram submetidos a uma avaliação do fundo de olho bilateral, sob midríase, utilizando o retinógrafo portátil acoplado a um smartphone e o retinógrafo comercial padrão (4 imagens por paciente). Dois oftalmologistas experientes avaliaram a qualidade de todas as imagens e atribuíram a elas uma pontuação entre 1 e 5, de acordo com a escala Likert. Os fatores relacionados a qualidade das imagens foram avaliados utilizando uma análise de regressão multivariada. Dois oftalmologistas determinaram de forma mascarada a relação da escavação/ cabeça do nervo óptico de cada imagem e a concordância intra e interobservador foi calculada.

**Resultados:**

Noventa e oito imagens de 49 pacientes foram utilizadas neste estudo para análise de qualidade. Dez imagens de cinco pacientes (quatro do retinógrafo comercial padrão e um do retinógrafo portátil acoplado a um smartphone) foram excluídas das análises de concordância devido à baixa qualidade das mesmas, mas foram considerados nas análises de qualidade. Dos cinco pacientes com imagens excluídas, quatro foram capturadas pelo retinógrafo comercial padrão e uma pelo retinógrafo portátil acoplado a um smartphone. As medianas (intervalo interquartil) da qualidade das imagens não apresentaram diferença estatística entre o retinógrafo portátil acoplado a um smartphone e o retinógrafo comercial padrão (4 [4-5] versus 4 [3-4] respectivamente, p=0.06). As imagens obtidas com o retinógrafo comercial padrão e o diagnóstico de opacidade de meios apresentou uma correlação negativa com a qualidade da imagem. As concordâncias intraobservador (ICC =0,82, p<0,001 e 0,83, p<0,001, para o examinador 1 e 2, respectivamente) e interobservador (ICC = 0,70, p=0,001 e 0,81, p<0.001, para o retinógrafo portátil acoplado a um smartphone e retinógrafo comercial padrão, respectivamente) foram excelentes e estatisticamente significativas.

**Conclusões:**

Nossos resultados sugerem que o retinógrafo portátil acoplado a um smartphone apresenta uma qualidade de imagem semelhante ao retinógrafo comercial padrão, com concordância significativa na análise da relação escavação-cabeça do nervo óptico. Além dos bons resultados apresentados, o retinógrafo portátil acoplado a um smartphone pode ser considerado uma alternativa portátil de baixo custo para documentação de retina em cenários futuros de telemedicina.

## INTRODUCTION

The impact of senile chronic diseases in Brazil is be coming increasingly important considering the current aging pattern of the Brazilian population^(1)^. Ocular diseases such as glaucoma, macular degeneration, and diabetic retinopathy, besides cataract, are the leading causes of blindness in individuals aged >50 years^(2)^. The increasing prevalence of these diseases reinforces the need for a diagnosis based on fundus examination in national health programs.

Unfortunately, eye health programs are often not well integrated with the health system^(3)^. Barriers to access healthcare are derived from the limitations associated with technologies, providers, geographical distances, as well as other cultural, cognitive, and behavioral differences among the health service users^(4)^.

Previous studies in India^(5)^ and Kenya^(6)^ demonstrated that handheld devices can be used for fundus documentation by non-ophthalmologists in areas lacking assistance for the detection of retinal diseases. Nevertheless, non-ophthalmologist healthcare workers could capture high-quality images in children screened for retinopathy of prematurity with a portable fundus camera. Their photographs were uploaded and remotely graded for retinopathy of prematurity by a retina specialist with good sensitivity and specificity levels^(7)^. All recent advances in mobile devices that may facilitate telemedicine strategies are believed to improve the integration of eye healthcare system in countries with low resources. With this background, the purpose of the present study was to validate a new smartphone, handheld fundus camera (SHFC) by evaluating both the image quality and its agreement with those from a commercial fundus camera (CFC) in determining the cup-to-disc ratio in a cohort of patients.

## METHODS

### Ethics approval

This cross-sectional study was approved by the Research Ethics Committee of the PIO XII Foundation, Barretos, SP and by the Research Ethics Committee of *Hospital das Clínicas da Faculdade de Medicina de Ribeirão Preto,* SP. The norms of the Declaration of Helsinki and the International Conference on Standardization Note for Guidance on Good Clinical Practice (ICH, Topic E6, 1995) were followed. All subjects received extensive and detailed oral and written explanations of all project-related events, and they provided with their signed informed consent form.

### Participants

A total of 50 patients who were previously scheduled for eye examination by independent ophthalmologists from the ophthalmologic service of the AME, Barretos (São Paulo, Brazil) were included in this study.

The following were the inclusion criteria for the study subjects: age ≥18 years, no cognitive disability, the ability to undertake all necessary examinations, and no previous eye surgery performed in the last 2 months.

### Device development

The SHFC was developed using an optical system that could generate high-resolution images within the 45 degree of the fundus view. It was attached to a smartphone (Samsung Galaxy S7; 12-Megapixel camera resolution, 2.6-Megapixel image resolution; Samsung Electronics Co., Suwon, Korea) and its processor, display, global positioning system, and internet access for handling patient, examinations, and data reports were used. The safety of light exposure was previously compared with reference to the energy levels necessary for performing colored fundus imaging between SHFC and other CFC (Topcon retinal camera, 16.2-Megapixel camera resolution, 1.45-Megapixel image resolution; Topcon Healthcare Solutions, Oakland, USA). The light measurements were made in two moments: a preview mode, with continuous homogeneous illumination and a capturing mode, with an instantaneous flash power. All measurements were performed using a handheld power/energy meter (Vega, Ophir Photonics, Newport Co., Jerusalem, Israel) coupled with a thermopile-based laser energy sensor (Model 3A Ophir Photonics; Newport Co., Jerusalem, Israel). The distance between the camera optical system and the sensor was determined based on the optical alignment to ensure that all light from the devices would reach the sensor-sensitive area to enable detection of the highest optical power value. Ten measurements each were taken in both the preview and the capturing modes.

### Procedures

All patients scheduled for fundus imaging received at least one prior comprehensive ophthalmological evaluation, performed by the attending ophthalmologists from AME-Barretos, São Paulo, Brazil. On the scheduled day, the patients received one drop of 1% tropicamide and 10% phenylephrine for pupil dilation. After 25 min, a series of 3 photographs, followed by a series of 4 photographs of each eye, were captured by 2 trained nurses in a bright room by using the CFC and the SHFC, separately. The examiners had previously undergone three separate 1-h trainings. A masked examiner selected the best fundus picture of each eye from all patients, captured with both the devices.

The photographs of the anterior segment of both the eyes were also captured, which served as the measurement of the horizontal pupil diameter. The summarized demographic data (such as age and gender) and the individual spherical equivalent and diagnosis (such as glaucoma and suspects, retinal diseases, refraction errors, and cataract) were recorded on the same day and then subsequently analyzed. These data were collected from a summary review of the patients’ medical record, as the comprehensive ophthalmological evaluation was not directly performed by the researchers.

Two experienced, masked ophthalmologists evaluated all the fundus images on the same 19-inch LCD computer monitor. Each examiner graded an individual image based on the quality score with reference to the Likert 1-5 scale: 1 = Poor, unsatisfactory, or impossible to capture; 2 = Regular or partially satisfactory; 3 = Good or satisfactory; 4 = Very good or quite satisfactory; and 5 = Excellent or totally satisfactory.

The examiners also evaluated the randomly assorted individual images and attributed the values to the vertical cup/disc ratio by using a double-masked database of images (for both patient identification and the device used).

### Statistical analysis

Variables were described using the mean, median, standard error, 95% confidence interval (95% IC), and frequencies, as necessary. Images with insufficient quality (score <2) were excluded from the agreement analysis, but were included for quality comparisons (using the nonparametric Friedman’s 2-way analysis of variance by ranks). A linear mixed-effects multivariate regression was also performed to identify the factors associated with the image quality, as follows: device type, ocular diagnosis, pupil diameter (after mydriasis), spherical equivalent, and age.

In addition, an intraclass correlation coefficient (ICC) was used to assess interobserver agreement (for each device) as well as the agreement between devices (for each observer) in combination with the Bland-Altmann plot analysis, displaying the mean difference ± limits of agreement (±1.96 × standard deviation) of the vertical cup-to-disc ratio between the two devices. All analyses were performed using the Stata software (Stata 14.2; StataCorp LLC, Texas, USA). Statistical significance was set at p<0.05.

## RESULTS

The results of ocular safety when exposed to the SFHC are depicted in table 1. The mean optical power (0.14 ± 0.02 mW) and the mean radiant flash energy (0.29 ± 0.02 mJ) of the SHFC were found to be significantly smaller than those of CFC (0.50 ± 0.03 mW and 6.40 ± 0.05 mJ, respectively; p<0.001). Based on its presented lighting levels, the SHFC device was classified in Group 1 (safe) according to both the ISO 10940 and 15004-2 standards. Accordingly, 98 eyes of 49 patients (33 women [67.3%], mean age: 62.1 ± 10.2 years) were included in this study. Table 2 displays the summarized demographic data and diagnoses.

**Table 1:** Illumination levels for color fundus imaging with the SHFC and CFC for retinal documentation

Device	Optical power (Preview mode)		Radiant flash energy (Capturing mode)	
	Mean ± SD	Maximum value	Mean ± SD	Maximum value
SHFC	0.14 ± 0.02 mW	0.28 ± 0.03 mW	0.29 ± 0.02 mJ	0.29 ± 0.02 mJ
CFC	0.50 ± 0.03 mW^*^	14.50 ±0.05 mW^*^	6.40 ± 0.05 mJ^*^	46.00 ±0.10 mJ^*^

CFC 0.50 ± 0.03 mW^*^ 14.50 ± 0.05 mW^*^ 6.40 ± 0.05 mJ^*^ 46.00 ± 0.10 mJ^*^ SHFC= Smartphone-based Handheld Fundus Camera; CFC= Commercial Fundus Camera; SD= Standard Deviation;

* = p<0.001 for the paired comparison with SHFC results.

**Table 2:** Demographics of the participants included in the study.

	Age	Gender		
**Diagnosis**	(years)	**Female**	**Male**	**Total**
Glaucoma/suspects		14 (28.5%)	9 (18.3%)	23 (46.9%)
Retinal diseases	64.2 ± 11.3	14 (28.5%)	5 (10.2%)	19 (38.7%)
Refractive errors	52.8 ± 12.0	4 (8.2%)	2 (4.1%)	6 (12.2%)
Cataract	59.0	1 (2.0%)	0	1 (2.0%)
Total	62,1 ± 10.2	33 (67.3%)	16 (32.6%)	49 (100%)

One patient was excluded from all data analyses due to the loss of his images that were captured with the CFC. Another 5 patients were later excluded from the agreement analyses (for the cup-to-disc ratios; n=44 patients, 88 eyes) due to the poor quality of the acquired images (grade 1 or 2), but they were considered in the quality analyses. Of these five unclassifiable patient images, four were acquired with the CFC device and one with the SHFC device.

The main difficulties reported during the SHFC examinations included the following: manual centralization of the preview image, patient collaboration, pupil size, eye alignment, and screen handling. These reports, however, were not objectively analyzed.

**Figure 1:**
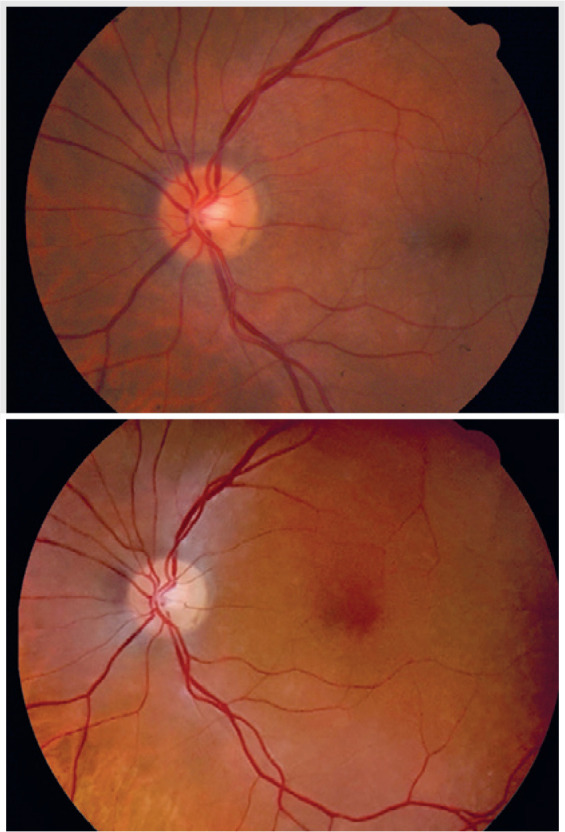
Examples of fundus images captured from the same patient using the two different devices. On top, an image of participant #27 acquired with the CFC. On the bottom, an image from the same participant acquired with the SHFC.

Figure 1 illustrates examples of retinal images captured by the same individual using the two different devices. The median [interquartile interval] score for image quality was 4 [4-5] for the SHFC, and it did not differ from the corresponding values for the CFC (4 [34]; p=0.06). Thus, we observed that the use of the CFC device and the diagnosis of “cataract” had a significant negative correlation with the image quality (Table 3).

**Table 3:** Multivariable regression analysis with mixed-effects model of factors associated with image quality

	Coefficient	SE	95% CI	p value
A. CFC^*^	-0.33	0.09	-0.51:-0.15	**0.001**
B. Pupil diameter	-0.02	0.08	-0.18:0.13	0.74
C. Spherical equivalent	0.10	0.07	-0.03:0.24	0.13
D. Age	-0.01	0.01	-0.03:0.01	0.31
E. Diagnosis^**^				
1. Retinal diseases	-0.22	0.27	-0.74:0.30	0.41
2. Cataract	-1.17	0.58	-2.30:-0.02	**0.04**
3. Refractive errors	0.16	0.32	-0.47:0.80	0.61

* = In comparison to the SHFC device;

** = In comparison to the diagnosis of glaucoma and suspects.

SE= Standard Error; 95% CI: 95% confidence interval.

**Figure 2:**
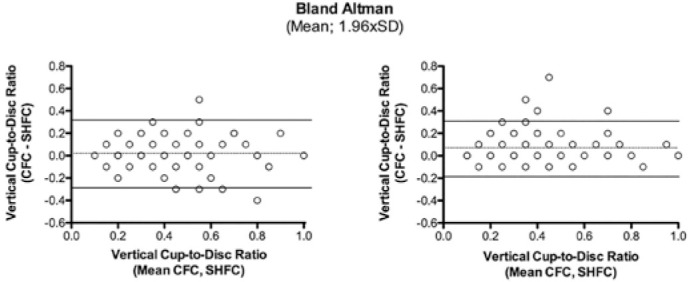
Bland-Altmann plots depicting the agreement analyses for the assessment of the vertical cup-to-disc ratios between the two devices for examiner 1 (left panel) and examiner 2 (right panel). Dashed lines represent the mean difference in the values between the devices (CFC-SHFC) and the continuous lines represent the limits of agreement calculated with ±1.96 × Standard Deviation (SD).

The interobserver agreement for the evaluation of the cup-to-disc ratio was considered “good” with the SHFC images (ICC=0.70; p=0.001) and “very good” with the CFC images (ICC=0.81; p<0.001). The agreement coefficients between the devices were “very good” (examiner 1: ICC=0.82; p<0.001, examiner 2: ICC=0.83; p<0.001) (Table 4). The Bland-Altmann plot analyses displayed a good agreement between both the devices for both the examiners. However, data for examiner 2 showed a higher mean difference in the vertical cup-to-disc ratio (mean difference ± limit of agreement: 0.02 ± 0.29 and 0.07 ± 0.25 for examiners 1 and 2, respectively), and four images had differences of >0.3 (Figure 2).

**Table 4:** Intraclass correlation analysis of the agreement between devices for each examiner with regards to the cup-to-disc ratio results

Examiner	ICC	95% CI	pvalue
1	0.82	0.73:0.87	<0.001
2	0.83	0.75:0.88	<0.001

## DISCUSSION

Recent reports estimate that at least 2.2 billion people will present visual impairment, with age-related macular degeneration, glaucoma, and diabetic retinopathy being the most common causes, besides cataract and refractive error^(8-12)^. Latin America and the Caribbean countries could have saved $ 6,281 million by 2020 if the blindness prevention programs had been implemented in the past 13 years^(13)^. These data reinforce the need for an integrated health system that is capable of early detection of these conditions and the prevention of blindness at lower costs. Portable equipment compatible with telemedicine can facilitate low-cost integration of the health system against the burden of ocular diseases^(14)^.

Among a few others, the prototype used in the present study may be considered as a safe alternative for developing a telemedicine-based system for the detection of ocular diseases integrated with the health system. This system can be considered safe owing to its at least 3-times lower optical power than that of the CFC in a preview mode and 22-times lower power in a capturing mode, in addition to its affordability (estimated to be US$ 5,000, which is 6-times lower than that for most other available table retinal cameras).

Regarding the quality of images captured, a significant superiority was noticed in the images captured by the SHFC in comparison to those by CFC, since the multivariate analysis indicated a significant correlation with the device type (SHFC), besides the presence of cataract. The other parameters analyzed (including age, pupil diameter, retinal diseases, and refractive errors) presented no significant association with the image quality (Table 3). A significant negative association was noted between cataract and image quality (p=0.04), however, this point needs to be considered along with the fact that only one patient with this diagnosis was included in the study, which may potentially affect further conclusion.

The clinical validation of the SHFC was verified through the determination of the cup-to-disc ratios in the fundus images in comparison to the CFC across two masked examiners. First, our results showed good significant agreement between the observers for both the devices, with the coefficient being higher for the CFC device (ICC=0.81; p<0.001) than for the SHFC device (ICC=0.70; p=0.001). It is believed that the interobserver agreement in the clinical evaluation of the optic nerve varies, but it can be higher if the analysis is based on retinal images of the fundus^(14,15)^.

Second, the Bland-Altman plots showed good levels of agreement. Moreover, examiner 2 may have performed worse than examiner 1 considering the four images with the vertical cup-to-disc ratio differences between the devices being >0.3 in his evaluation. One of the examiners is a glaucoma specialist, while the other is a general practice ophthalmologist, both with several years of clinical experience. We thus speculated that the difference in the background of the two examiners indirectly accounted for the higher variability presented as well as for the potential differences in the generation of some images by the different devices. Thus, good ICC values recorded herein accounts for the higher credibility of both the examiners, although examiner 2 demonstrated higher variability during his evaluation.

The intraobserver agreement in determining the cup-to-disc ratio was also good and significant for each examiner (ICC = 0.82; p<0.001 versus 0.83; p<0.001). These consistent results of comparable performance between the devices in producing reliable fundus image (at least for the good determination of the optic nerve excavation boundaries) account for the clinical validation of SHFC, despite the technical differences between them.

Previous studies have evaluated both the interobserver and the intraobserver agreements in cup-to-disc ratios measured in the fundus images captured by different devices. The present results are similar to those described for both the interobserver (0.67-0.9) and intraobserver (0.79-0.92) agreement levels in some past studies^(14,16,17)^. However, only Shuttleworth et al.^(17)^ presented their coefficient results as ICC, with no comparison between two different devices. Waisbourd et al.^(18)^ reported lower ICC levels of agreement (ICC = 0.71 for the intraobserver and ICC = 0.69 for the interobserver agreement) with the use of a handheld fundus camera, but they did not compare the data with those of other routinely used devices. On the other hand, Miller et al.^(19)^ compared the performance of a non-mydriatic handheld fundus camera to that of a conventional tabletop mydriatic camera and observed slightly lower k values for both the intraobserver (0.64) and interobserver (0.54) agreements. Thus, our good results validate and indicate the potential clinical applications of the SHFC.

**Figure 3:**
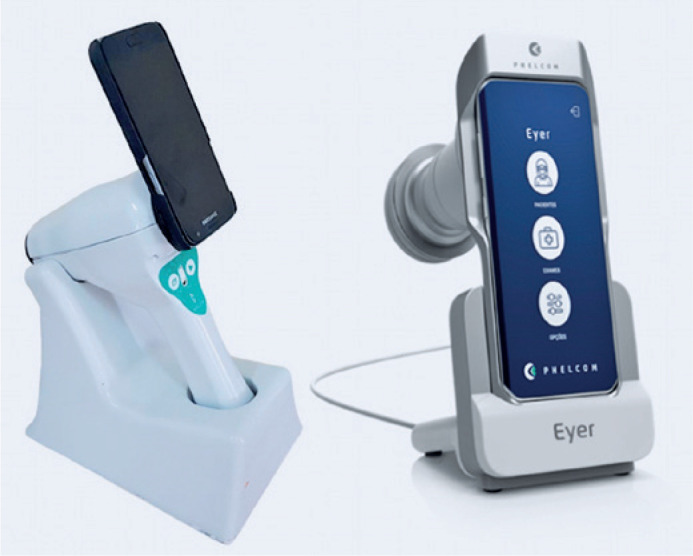
Photographs of the prototype used in the study (left panel: SHFC) and that of its latest commercial version (right panel: *Eyer*).

The present study, however, has some limitations. First, we included no healthy control group. Our study protocol was applied for the evaluation of all cup-to-disc ratios, because it was not designed for glaucoma diagnosis. In addition, both patients with glaucoma and suspects were included in the regression analyses. Second, despite patients and examiners reporting great comfort with the SHFC device at the very first tests, the factors of comfort and ease of handling were not objectively analyzed. Finally, a new non-mydriatic version of the SHFC (Figure 3) is now commercially available, but it was not tested in the present study.

Several portable retinal imaging devices have emerged in the past few years as alternatives for better-integrated eye health care. We believe that an ideal portable device for fundus image should be light-weighted, easy to handle, of low-cost, non-mydriatic, and equipped with the facility of data transfer. Several available portable devices meet some of these features, and the new version of the prototype presented in this study represents an option that fulfills all of them. Further research is warranted to determine the proposed device’s capability to generate good-quality images without mydriasis and to validate its sensitivity and specificity levels for the diagnosis of the most prevalent ocular diseases, as a possible new alternative to the telemedicinal approach for the future.
